# High‐fat diet‐induced dysbiosis mediates MCP‐1/CCR2 axis‐dependent M2 macrophage polarization and promotes intestinal adenoma‐adenocarcinoma sequence

**DOI:** 10.1111/jcmm.14984

**Published:** 2020-01-19

**Authors:** Tianyu Liu, Zixuan Guo, Xueli Song, Li Liu, Wenxiao Dong, Sinan Wang, Mengque Xu, Cheng Yang, Bangmao Wang, Hailong Cao

**Affiliations:** ^1^ Department of Gastroenterology and Hepatology General Hospital Tianjin Medical University Tianjin Institute of Digestive Disease Tianjin China; ^2^ Department of Gastroenterology Sir Run Run Shaw Hospital School of Medicine Zhejiang University Hangzhou China; ^3^ State Key Laboratory of Medicinal Chemical Biology and College of Pharmacy Nankai University Tianjin China; ^4^ Tianjin International Joint Academy of Biomedicine Tianjin China

**Keywords:** gut microbiota, high‐fat diet, intestinal carcinogenesis, MCP‐1/CCR2 axis, tumour‐associated macrophages

## Abstract

High‐fat diet (HFD) is a well‐known risk factor for gut microbiota dysbiosis and colorectal cancer (CRC). However, evidence relating HFD, gut microbiota and carcinogenesis is limited. Our study aimed to demonstrate that HFD‐induced gut dysbiosis promoted intestinal adenoma‐adenocarcinoma sequence. In clinical study, we found that HFD increased the incidence of advanced colorectal neoplasia (AN). The expression of monocyte chemoattractant protein 1 (MCP‐1), CC chemokine receptor 2 (CCR2) and CD163 in CRC patients with HFD was significantly higher than that in CRC patients with normal diet. When it comes to the *Apc^min/+^* mice, HFD consumption could induce gut dysbiosis and promote intestinal carcinogenesis, accompanying with activation of MCP‐1/CCR2 axis that recruited and polarized M2 tumour‐associated macrophages. Interestingly, transfer of faecal microbiota from HFD‐fed mice to another batch of *Apc^min/+^* mice in the absence of HFD could also enhance carcinogenesis without significant body weight gain and induced MCP‐1/CCR2 axis activation. HFD‐induced dysbiosis could also be transmitted. Meanwhile, antibiotics cocktail treatment was sufficient to inhibit HFD‐induced carcinogenesis, indicating the vital role of dysbiosis in cancer development. Conclusively, these data indicated that HFD‐induced dysbiosis accelerated intestinal adenoma‐adenocarcinoma sequence through activation of MCP‐1/CCR2 axis, which would provide new insight into better understanding of the mechanisms and prevention for HFD‐related CRC.

## 1 INTRODUCTION

Colorectal cancer (CRC) is one of the most common cancers and the leading cause of cancer deaths worldwide.^1^ Apart from hereditable components, environmental factors including high‐fat diet (HFD) could be considered the major factor in susceptibility to CRC.^2^ But the underlying mechanisms are still at large unknown. Human colon homes to diverse communities of microbiota that contribute to the health and disease, and the composition of gut microbiota is known to vary, depending on host genetics, hygiene, diet and other factors.^3^, ^4^, ^5^ Lately, the effects of HFD on gut microbiota dysbiosis are widely concerned.^6^ Dysbiosis is defined as a disturbance in the microbiome structure, such as loss of beneficial microorganisms, and expansion of pathological microorganisms, promoting the development of CRC.^7^, ^8^ However, the specific mechanisms that how HFD‐induced dysbiosis affects intestinal carcinogenesis remain to be clarified.

Tumour microenvironment involved in the development of cancer. Chemokines recruit immune cells to inflamed sites and contribute to the development of CRC.^9^ Monocyte chemoattractant protein 1 (MCP‐1), also known as chemokine C‐C motif chemokine ligand 2 (CCL2), is a member of the C‐C chemokine family and a potent chemotactic factor for monocytes. MCP‐1 functions through its receptor CC chemokine receptor 2 (CCR2).^10^, ^11^ Monocytes which recruit to tumour microenvironment through the MCP‐1/CCR2 axis are polarized to M2 tumour‐associated macrophages (TAMs), contributing to tumour progression.^9^ Importantly, the expression of MCP‐1 was related to the number of TAMs and stage of CRC.^12^ However, how the dysbiosis induced by HFD affects tumour microenvironment and TAMs has not yet elucidated.

A recent study has reported HFD‐mediated dysbiosis promoted tumour progression in the small intestine in *K‐ras^G12Dint^* mice.^13^ This is a well‐characterized serrated hyperplasia model. However, about 80%‐90% of sporadic colorectal neoplasms followed *adenomatous polyposis coli* (*APC*) gene mutation pathway. The *APC* gene was defined as the ‘‘gatekeeper’’ of colonic carcinogenesis. *APC* mutation leads to intestinal carcinogenesis along the adenoma‐adenocarcinoma sequence ultimately to invasive cancer, and this is more in line with the progression of CRC *Apc^min/+^* mice which carried a germline mutation at codon 850 of the *Apc* gene and spontaneously developed intestinal adenoma were used in our study.^14^, ^15^, ^16^, ^17^ In addition, we explored the mechanism of innate immunity and the role of microbiota different from previous study.

The current work showed that HFD increased the incidence of advanced colorectal neoplasia (AN) and activated the MCP‐1/CCR2 axis in CRC patients with HFD in daily life. We further provided the evidence that HFD‐induced gut dysbiosis stimulated tumour cell proliferation and decreased apoptosis, modulated cytokines and chemokines by activating MCP‐1/CCR2 axis and ultimately promoted intestinal carcinogenesis. Faecal microbiota transplantation (FMT) study and antibiotics treatment further supported the role of gut microbiota in tumour development. Accordingly, these findings will provide new insights into better understanding of the mechanisms of HFD‐related CRC and highlighting a potential therapeutic strategy.

## 2 MATERIALS AND METHODS

### 2.1 Study population and diets

A retrospective cohort study was adopted to investigate HFD in relation to AN which was defined as adenoma ≥1 cm, adenoma with villous component or high‐grade dysplasia (HGD) or invasive carcinoma. The subjects comprised 2338 individuals who underwent a colonoscopy at the Digestive Endoscopy Center of Tianjin Medical University General Hospital, Tianjin, China, from January 2016 to August 2018. Participants were divided into HFD group and control group according to registration form before colonoscopy. HFD was defined as the average daily intake of red meat exceeding 100 g in the past year. Control diet was defined as the average daily intake of red meat less than 100 g in the past year.^18^ Then, we randomly selected 30 CRC patients with no significant differences in pathological characteristics (TNM classification) from the HFD group (n = 15) and the normal diet group (n = 15) for IHC staining to evaluate the difference of MCP‐1(bs‐1955R, Bioss), CCR2 (bs‐0562R, Bioss) and M2 TAMs (CD163) (ab182422, Abcam) expression. In addition, we selected 40 human colorectal tissue specimens (10 non‐neoplastic colon tissues, 10 adenomas (low‐grade dysplasia [LGD]), 10 adenomas (HGD) and 10 carcinomas) to evaluate the expression of MCP1, CCR2 and CD163 during the normal‐adenoma‐adenocarcinoma sequence by IHC staining. Informed consents were signed by all patients, and ethical approval was obtained from the Ethics Committee of General Hospital, Tianjin Medical University, China.

### 2.2 Mice and treatment

Four‐week‐old *Apc^min/+^* mice were randomized into control group (control diet: 16% fat content, 20% protein content and 64% carbohydrate content, Table S1) and HFD group (HFD: 60% fat content mainly composing of lard and soybean oil, 20% protein content and 20% carbohydrate content, Table S1) and housed for 12 weeks under specific pathogen‐free environment. Secondly, we performed FMT. Fresh faecal pellets from HFD mice and control mice were suspended in PBS (0.1 g/1 mL), centrifuged for 5 minutes at 800 *g* and collected the supernatant. Two groups of four‐week *Apc^min/+^* mice were treated with streptomycin (20 mg) for 3 days to eliminate the native gut microbiota, and then, 300 μL of the collected supernatant was transplanted to mice based on the previous studies.^19^ The transfer experiment was carried out for 8 weeks and inoculated 16 times (seven times in the first week, once every other day in the second week and once per week in the last 6 weeks). All the mice were fed with control diet. Thirdly, we performed antibiotics treatment to further verify the causal relationship between gut dysbiosis and intestinal carcinogenesis. HFD with a daily dose of antibiotics cocktail (500 mg ampicillin, 250 mg vancomycin, 500 mg neomycin and 250 mg metronidazole) was provided to a new batch of *Apc^min/+^* mice (HFD‐Abx group) for 12 weeks. Antibiotics mixture was added to drinking water.^20^, ^21^, ^22^ All animal experiments were conducted under the protocol of the Institutional Animal Care and Use Committee at Tianjin Medical University, Tianjin, China.

### 2.3 Intestinal processing

Mice were killed after 12 weeks, and caecal contents were immediately collected. Swiss‐rolled terminal small intestine and colon were fixed in 10% neutral‐buffered formalin and then were made into paraffin‐embedded tissue sections for haematoxylin and eosin or immunohistochemical staining. Adenomas excised from proximal and middle small intestinal were immediately frozen in liquid nitrogen and then stored at −80℃ for subsequent analysis.

### 2.4 Pathological analysis

The formalin‐fixed intestinal tissues were routinely dehydrated, equilibrated and embedded in paraffin. LGD and HGD refer to morphological abnormalities that occur in the lower half and upper half of the epithelium, respectively. Intramucosal carcinoma refers to the tumour invades into the lamina propria but not through the muscularis mucosae. For IHC staining, paraffin‐embedded intestine tissues were deparaffinized, rehydrated and incubated with primary antibodies overnight at 4℃. The slides were then incubated with a biotin‐labelled secondary antibody. The expression of Ki‐67 (ab16667, Abcam), MCP‐1 (ab9669, Abcam) and CCR2 (bs‐1671R, Bioss) was evaluated. Five fields were randomly observed for each slice to count the number of positive cells. Quantitative analysis to obtain the average percentage of positive cells in each tumour.

### 2.5 TUNEL assay

The apoptosis of tumour cells is assessed using the terminal deoxynucleotidyl transferase dUTP nick end labelling (TUNEL) method. Five fields were randomly observed for each slice to count the number of positive‐stained cells. At least three tumours were randomly selected from each mouse for counting statistics.

### 2.6 Immunofluorescent staining

Tissue sections were incubated with specific antibodies against F4/80 (total macrophages, ab6640, Abcam), iNOS (M1 TAMs, ab15323, Abcam) and MR (M2 TAMs, ab64457, Abcam) overnight. The sections were washed three times with 1× PBS and then incubated with fluorochrome‐conjugated secondary antibody for 60 minutes in the dark. DAPI (4, 6‐diamidino‐2‐phenylindole, blue, Southern Biotech) was finally added to the sections and incubated for 5 minutes. The fluorescence microscope (Lycra) was used to observe and photograph the images.

### 2.7 Real‐time quantitative PCR analysis

Total RNA was isolated using the RNeasy Mini Kit (Qiagen) and cDNA reverse transcription with TIANScript RT Kit (TIANGEN, Inc). The quantitative PCR was performed using the Power SYBR Green PCR Master Mix and carried out on a Step One Plus Real‐Time PCR System (Applied Biosystems). The oligonucleotide primers for target genes were shown in Table S2. The standard ΔΔCt method was used to evaluate the relative mRNA expression of each sample.

### 2.8 Western blot analysis

The tumour tissues from small intestine were lysed with RIPA buffer supplemented with proteinase inhibitor and phosphatase inhibitor (Sigma). After homogenization and centrifugation (12 000 *g*, 4℃, 15 minutes), the protein concentrations were determined using bicinchoninic acid protein assay (Thermo Scientific Inc). Proteins were separated and then transferred onto a PVDF membrane. The membranes were incubated sequentially with specific primary antibodies against MCP‐1 and CCR2 and horseradish peroxidase‐conjugated second antibodies. Proteins were quantified using image processor program (Image J).

### 2.9 Gut microbiota analysis

Amplification of the V3‐V4 region of the 16S rRNA transcripts was performed for faecal samples. Faecal DNA was obtained from frozen faeces using QIAamp DNA Stool Mini Kit (Omega Bio‐Tek). The collected amplicons were sequenced using 2 × 300 v3 sequencing chemistry on Illumina MiSeq platform. High quality reads were mapped into operational taxonomic units (OTUs, 97% identity) using Mothur (version v.1.30.1). Alpha (within a community) and beta diversity (between communities) were estimated according to the OTUs sequences. Principal component analysis (PCA) was carried out on the resulting matrix of distances. In addition, linear discriminant analysis of the effect size (LEfSe) was used to identify communities or species with significant differences.

### 2.10 Caecal short‐chain fatty acids (SCFAs) analysis

Caecal contents were diluted, acidified and filtered, and SCFAs were quantified by gas chromatography.^13^ The carbohydrates that escaped digestion in the small intestine entered the right colon, and the bacterial activity is higher here, so the SCFAs concentration is higher in the caecum and ascending colon, and gradually decreased in the right colon. The nitrogen was used as the carrier gas, and the velocity was controlled to 1 mL/min. The initial temperature of column temperature was 100 and maintained for 0.5 minutes. The temperature was then raised to 180℃ at a rate of 8℃/min for 1 minutes. The temperature was increased to 200℃ at 20℃/min and maintained for 5 minutes. The detector temperature was controlled at 240℃, and the injection volume was 1 μL. Quantitative determination of SCFAs concentrations with calibration curve method.

### 2.11 Statistical analysis

SPSS 22.0 (SPSS) was used for statistical analysis. All of the average values were presented as *mean* ± SD. Determination of statistical differences for multiple comparisons by one‐way *ANOVA* analysis. The paired Student's *t* test was used to define the difference between mean values. And the categorical variables were compared by chi‐square test or Fisher's exact test. *P* < .05 indicated significant statistical difference.

## 3 RESULTS

### 3.1 HFD increased the incidence of AN and activated MCP‐1/CCR2 axis in tumour tissues of CRC patients

**Figure 1:**
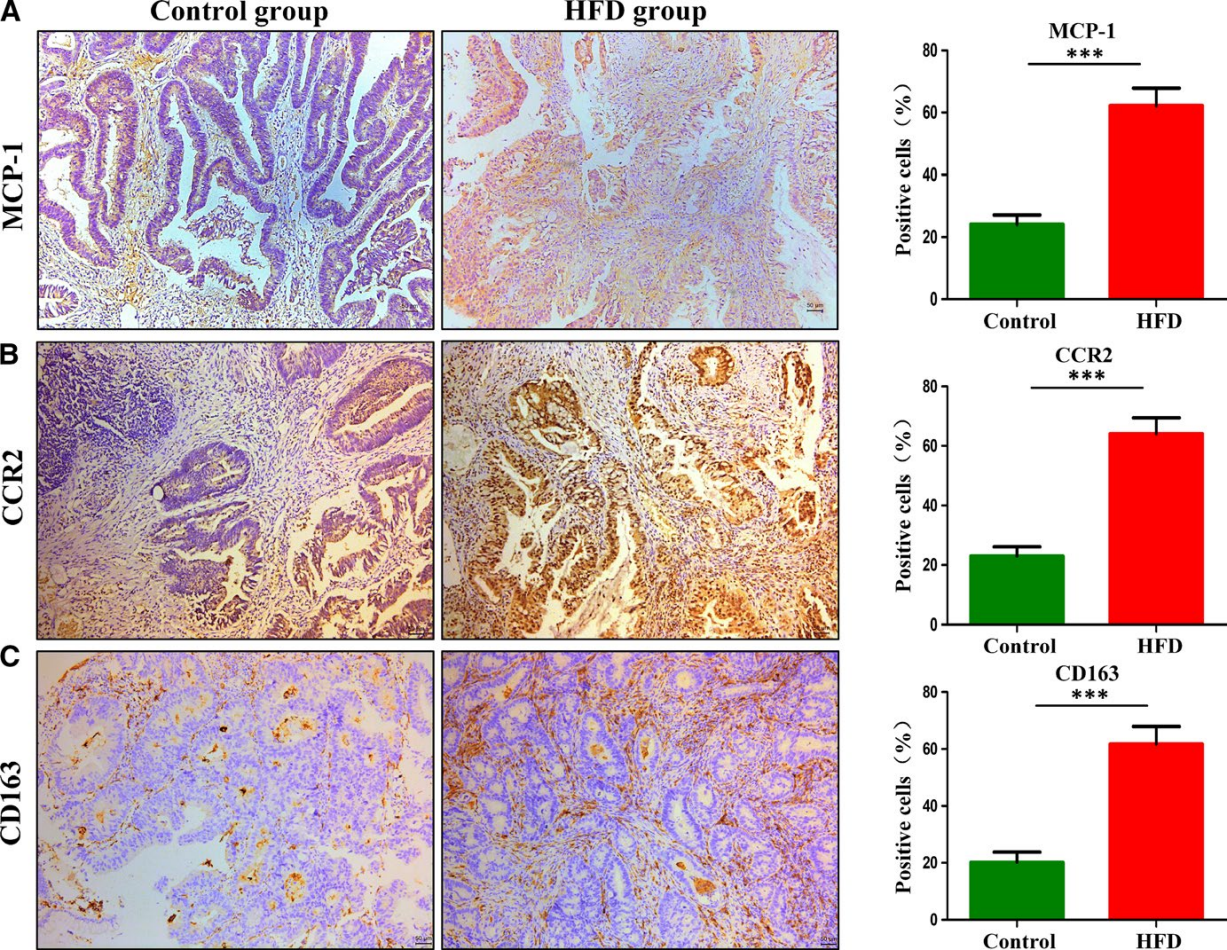
High‐fat diet up‐regulated the expression of MCP‐1 and CCR2 in human colorectal cancer tissues. A‐C, Immunohistochemistry analysis of CRC tissues. Scale bar: 50 μm. ***P < .001. CRC, colorectal cancer. Control, n = 15; HFD, n = 15

A total of 2338 individuals underwent colonoscopy were selected. They were further divided into the HFD group (n = 560, 24%) and the control group (n = 1778, 76%). In total, 55 (9.8%) patients with AN were detected in HFD group. The basic characteristics of patients were shown in Table 1. Participants with HFD were more likely to develop AN, especially invasive carcinoma. In addition, the intake of HFD could increase the malignant degree of adenocarcinoma. Then, we randomly selected 15 patients with CRC from each of the above two groups for IHC staining. The characteristics of 30 patients with CRC were shown in Table S3. BMI (kg/m^2^) ≥25 was defined as obesity. Current smoker was defined as smokers who smoked every day for the past 12 months; former smoker was defined as subjects no smoking at least 12 months before enrolment; never smoker was defined as subjects who had never smoked before. There were no significant differences in age, gender and other factors of the two groups. IHC staining showed that the expression of MCP‐1 and CCR2 in CRC patients with HFD was significantly higher than that in CRC patients with normal diet (Figure 1A‐B). Moreover, with the activation of MCP‐1/CCR2 axis, the M2 TAMs marker CD163 presented higher expression in the HFD group (Figure 1C). We then examined the expression of MCP‐1, CCR2 and CD163 in human colonic tissues and found that the expression of these proteins showed a gradual increase during intestinal normal‐adenoma‐adenocarcinoma sequence (Figure S1A‐C).

**Table 1:** High‐fat diet increased the incidence of advanced colorectal neoplasia

Characteristics	High‐fat diet (n = 560)	Control diet (n = 1778)	*P*
Male/Female	347/213	785/993	<.001
Age (y)	48.92 ± 1.39	54.1 ± 1.33	<.01
Smoking			.286
Current	93	273	.477
Former	39	97	.183
Never	428	1408	.165
Alcohol consumption (≥3 times/wk)	47	117	.1545
Tea consumption (≥3 times/wk)	156	477	.6627
Coffee consumption (≥3 times/wk)	37	116	.9222
Egg consumption (≥5 times/wk)	537	1690	.4941
Milk consumption (≥5 times/wk)	479	1486	.2899
Vegetable consumption (≥5 times/wk)	548	1756	.1536
Advanced neoplasia	55	125	<.05
Diameter ≥1 cm	33	79	.161
High‐grade dysplasia	10	21	.275
Villus adenoma	17	43	.421
Adenocarcinoma	17	30	<.05
TNM stage of cancer			<.05
Stage II	3	16	<.05
Stage III	5	7	.733
Stage IV	9	7	<.05

### 3.2 The experimental flow and body weight change during the study

All the treatment was tolerated by *Apc^min/+^* mice (Figure S2A,D). All mice were in good condition, and no mortality occurred throughout the treatment period. Mice with HFD showed mild blood in stools in the last 2 weeks. The body weight of HFD group was significantly higher than that of control group (Figure S2B‐C). There was no significant difference in body weight change between FMT‐C group and FMT‐H group (Figure S2E‐F). After antibiotics cocktail treatment, the body weight significantly decreased compared with HFD group (Figure S2B‐C).

### 3.3 HFD administration accelerated intestinal carcinogenesis

**Figure 2:**
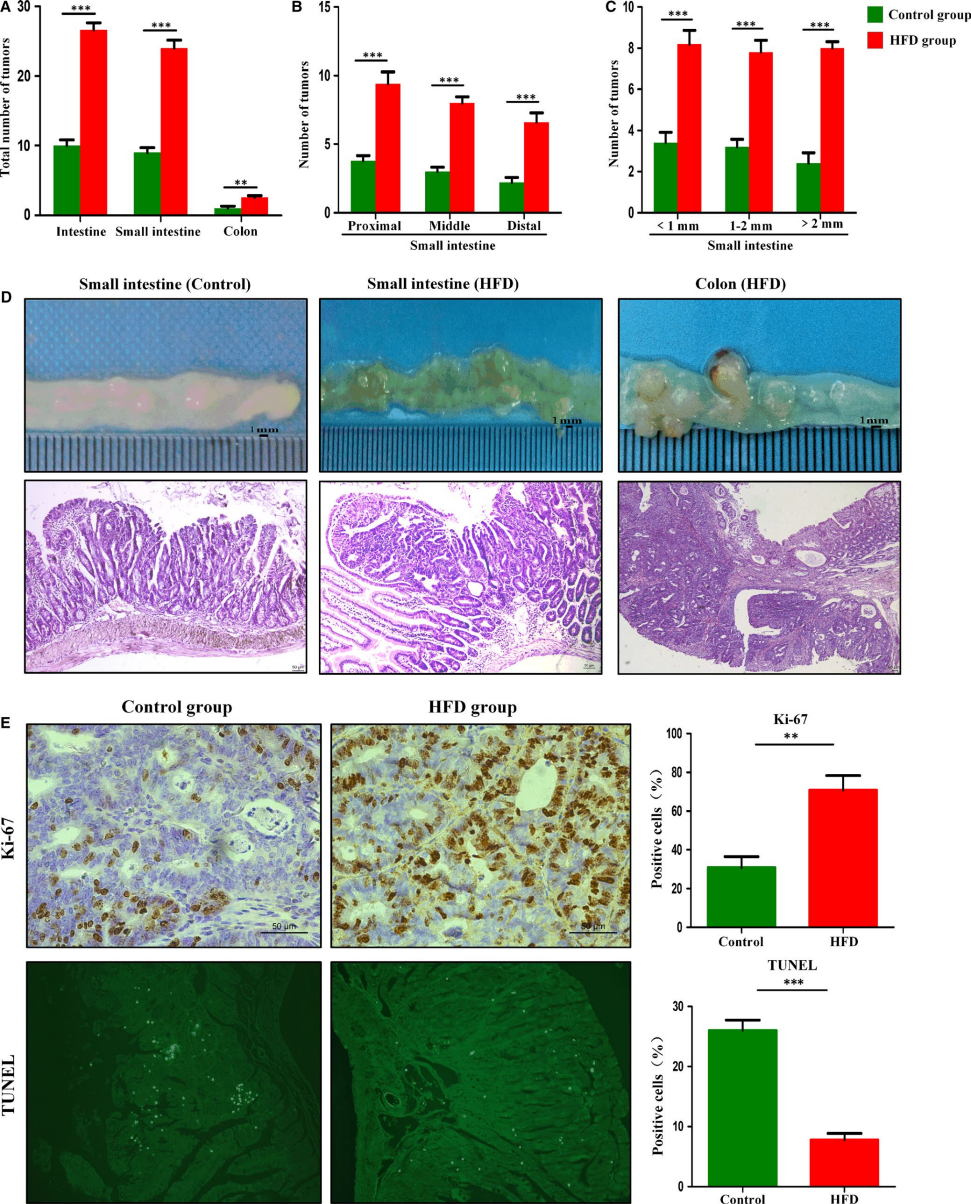
High‐fat diet accelerated intestinal adenoma‐adenocarcinoma sequence. A‐C, Tumour numbers of the HFD group and control group. D, The representative gross and histological appearance of intestinal tumours from the HFD group and control group were shown. E, Small intestinal sections from HFD‐treated and untreated *Apc^min/+^* mice were stained with Ki‐67 and TUNEL. Scale bar: 50 μm. **P < .01, ***P < .001. HFD, high‐fat diet. Control, n = 7; HFD, n = 8. HFD + Abx, n = 8

The total tumour number in HFD‐supplemented mice was significantly increased compared with that in the control group (Figure 2A). HFD promoted intestine tumour development in all the three segments of the small intestine (Figure 2B). Compared with the control group, the number of tumours of various sizes increased in the HFD group (Figure 2C). HGD, including intramucosal carcinomas, was detected in 75% (6/8) HFD‐fed *Apc^min/+^*mice, whereas adenomas with LGD were found only in 29% (2/7) of *Apc^min/+^* mice supplemented with control diet, and no dysplastic adenomas were found in the remaining 71% (5/7) mice (Figure 2D). The above results indicated that HFD accelerated intestinal malignancy independent of weight gain. Cell proliferation and apoptosis were used to assess the effects of HFD on intestinal tumour progression. The number of proliferating tumour cells was significantly increased in the HFD group compared with control group. The apoptotic cells of tumours were detected by TUNEL staining, and HFD group had less apoptotic cells in tumours than control group (Figure 2E). These findings suggested that HFD played a crucial part in tumour development by promoting proliferation and inhibiting apoptosis.

### 3.4 Gut dysbiosis involved in HFD‐induced tumorigenesis

**Figure 3:**
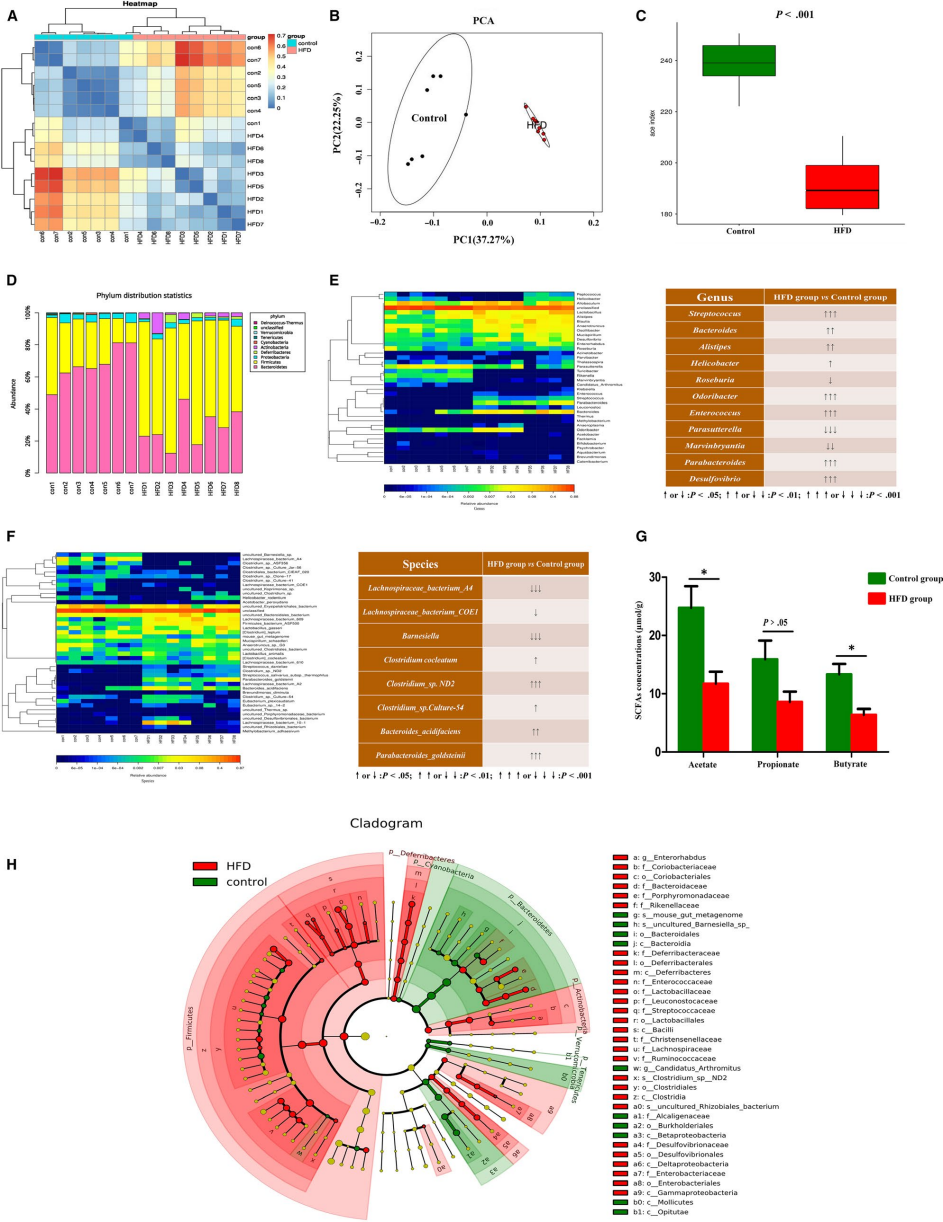
High‐fat diet‐induced gut dysbiosis during intestinal carcinogenesis. A, Beta‐diversity analysis showed a clear difference. B, Principal component analysis (PCA) of faecal bacteria in two groups. C, Ace index reflected the species richness of faecal samples. D‐F, The microbiota community composition between HFD group and control group was different at phylum, genus and species levels. G, The concentrations of SCFAs (Acetate and Butyrate) in caecal contents were significantly decreased after HFD treatment. H, Analysis of gut microbiota at different taxonomy levels and key bacteria changes during carcinogenesis. *P < .05. Control, n = 7; HFD, n = 8

The diversity of faecal microbial community strikingly altered after HFD feeding (Figure 3A). The result of the PCA showed that the luminal microbial community in HFD mice was significantly different from those in the control mice (Figure 3B). Richness estimators (Ace) showed the abundance of microbial in the HFD group significantly decreased compared with the control group (Figure 3C). In addition, cladogram was calculated by LEfSe, and the predominant bacteria of the microbiota in two groups were shown in Figure 3H *Bacteroides*, *Alistipes*, *Helicobacter* and *Enterococcus* were enriched in HFD mice. At phylum levels, the microbiota community composition between the two groups was different. The proportion of *Firmicutes* phylum in the control group was significantly less than *Bacteroidetes* phylum, whereas the proportion of *Firmicutes* phylum was greatly increased and significantly exceeded the *Bacteroidetes* phylum in the HFD group (Figure 3D). A heatmap of the microbial compositions at the genus level showed that HFD‐feeding mice had higher levels of opportunistic pathogens, such as *Desulfovibrio, Streptococcus*, *Parabacteroides* and *Odoribacter,* whereas the beneficial bacteria such as *Roseburia, Marvinbryantia* and *Parasutterella* were relatively less abundant in the HFD group compared with control group (Figure 3E).

### 3.5 HFD reduced the abundance of SCFAs‐producing bacteria and production of SCFAs

One of the main functions of bacteria in the colon is the fermentation of dietary fibre and resistant starch, resulting in the SCFAs production,^23^ which mainly composed of acetate, propionate and butyrate. SCFAs are suggested to serve as an energy source and have also been shown to reduce colonic inflammation, induce apoptosis, inhibit tumour progression and prevent CRC development.^24^, ^25^ Interestingly, at genus and species levels, the abundance of SCFAs‐producing bacteria in HFD group was significantly lower during the tumour development, such as *Roseburia*, *Barnesiella*, *Lachnospiraceae bacterium‐A4* and *Lachnospiraceae bacterium‐COE1* (Figure 3E‐F). The concentrations of acetate and butyrate in caecal contents also significantly decreased with the reduction of SCFAs‐producing bacteria in HFD mice (Figure 3G). The concentration of propionate in HFD group showed a downtrend compared with the control group (Figure 3G). These results showed that HFD decreased the content of SCFAs‐producing bacteria and reduced the production of SCFAs which might involve in the progression of intestinal tumour.

### 3.6 HFD‐induced dysbiosis activated the MCP‐1/CCR2 axis and promoted M2 TAMs polarization

**Figure 4:**
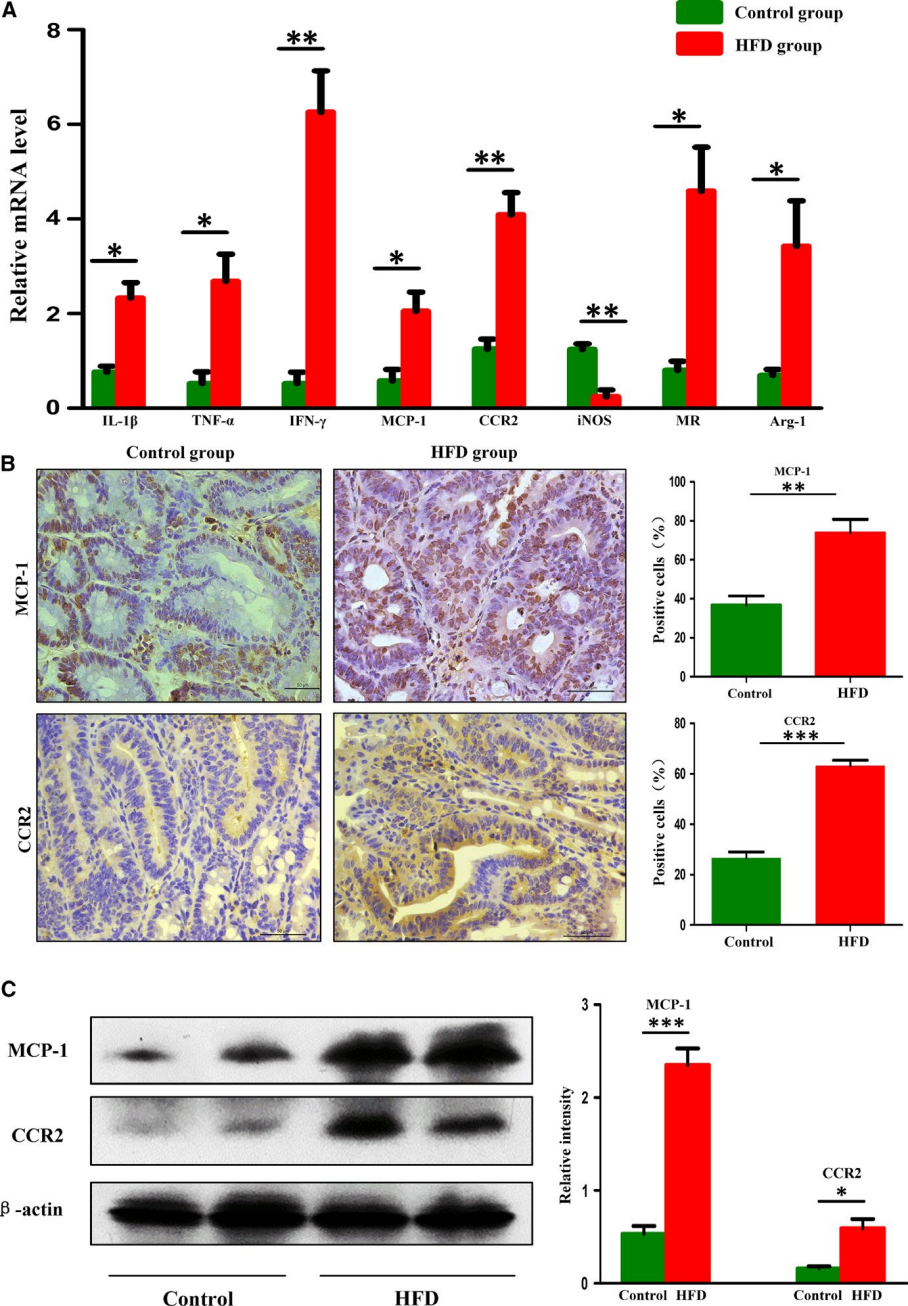
High‐fat diet‐induced dysbiosis activated MCP‐1/CCR2 axis. A, The inflammatory factors and TAMs mRNA levels in the small intestinal tumours. B‐C, Immunohistochemistry staining and Western blot results showed that the protein expression levels of MCP‐1 and CCR2 in intestinal tumours were significantly increased in HFD group. Scale bar: 50 μm. *P < .05, **P < .01, ***P < .001

**Figure 5:**
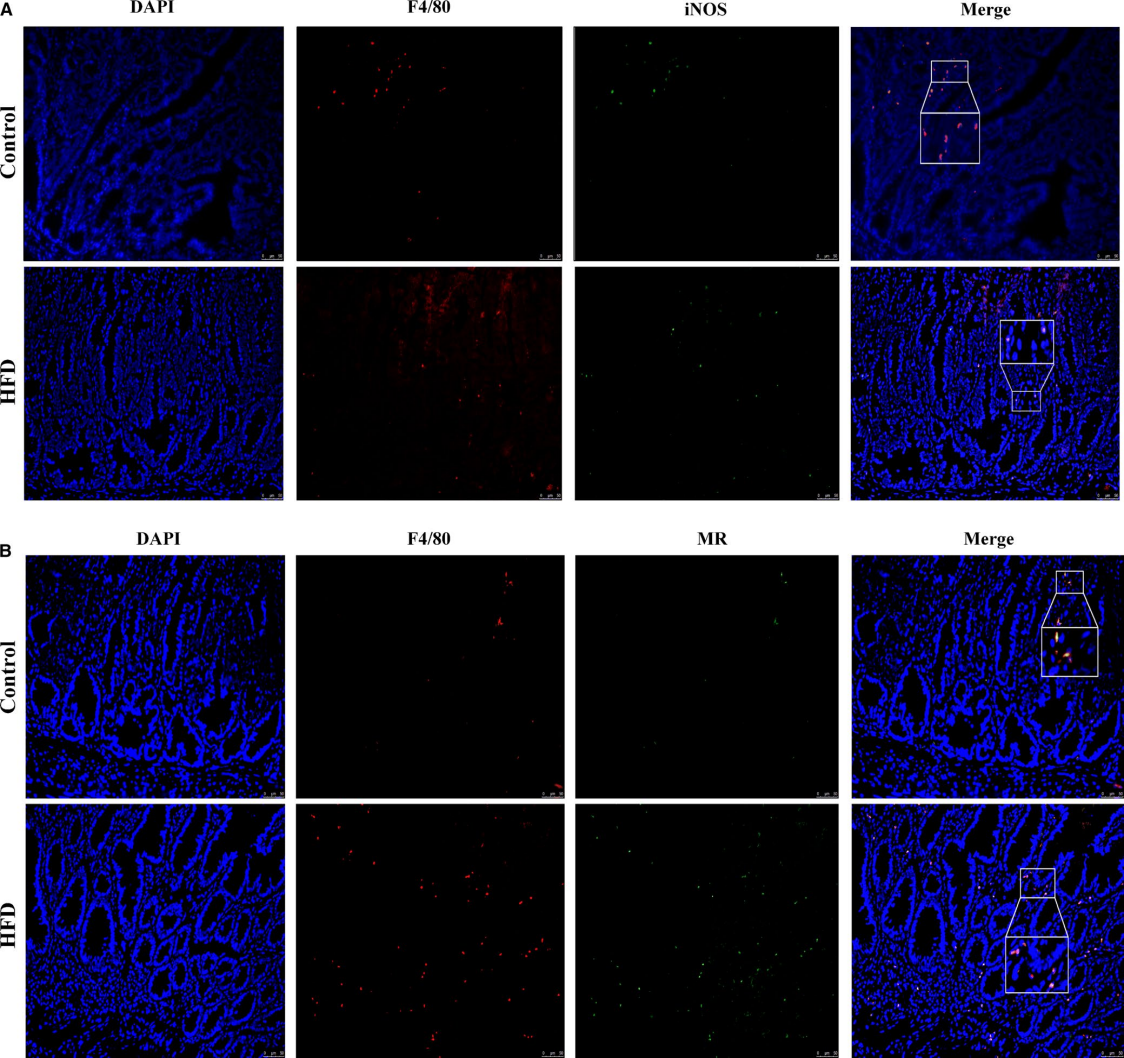
A‐C, Immunofluorescence assay suggested that the activated MCP‐1/CCR2 axis promoted M2 tumour‐associated macrophages recruitment and polarization. Scale bar: 50μm

Ample evidence supports the low‐grade inflammation involved in the initiation, promotion, invasion and metastasis of cancer.^26^, ^27^ We found that the mRNA expression of colonic inflammation mediators (IL‐1β, TNF‐α and IFN‐γ) was up‐regulated by HFD (Figure 4A). Recent studies suggest that MCP‐1/CCR2 axis plays a role in colon carcinogenesis.^11^, ^28^, ^29^, ^30^ In the present study, HFD exposure increased the mRNA expression of MCP‐1 and CCR2 (Figure 4A). The positive expression of MCP‐1 and CCR2 in intestinal tumours was enhanced by HFD supplementation (Figure 4B). Western blot analysis of MCP‐1 and CCR2 was consistent with the above results (Figure 4C). Macrophages were originated from blood monocytes and differentiated into distinct macrophage types, classically activated type (M1 TAMs) and alternatively activated type (M2 TAMs). M1 TAMs can help the host resist to the invasion of viral and microbial to fight against tumours,^31^ whereas M2 TAMs had pro‐tumoural functions which can promote tumour cell survival, proliferation and dissemination.^32^ The mRNA levels of M2 genes (MR and Arg‐1) were markedly elevated in the tumour tissues of HFD group, which was contrary to the expression of M1 gene (iNOS; Figure 4A). Immunofluorescent indicated that the number of total macrophages (F4/80) and M2 TAMs (MR) in HFD group were significantly higher than those in control group, whereas M1 TAMs (iNOS) of HFD mice presented a decreasing trend (Figure 5A‐B). These results suggested that the activated MCP‐1/CCR2 axis promoted M2 TAMs recruitment and polarization during the development of intestinal tumours after HFD treatment.

### 3.7 Faecal microbiota from HFD‐fed *Apc^min/+^* mice promoted intestinal carcinogenesis

**Figure 6:**
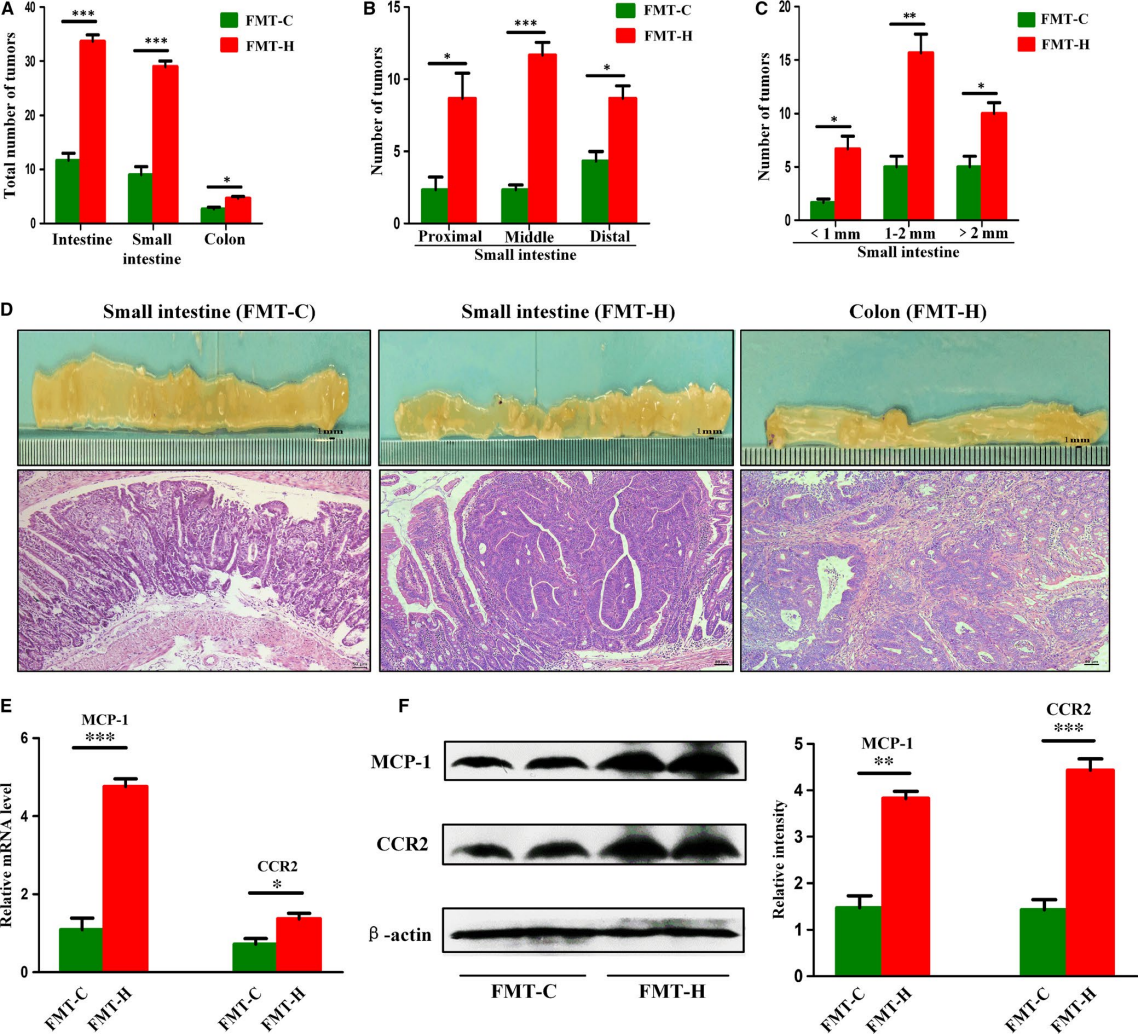
Faecal microbiota from high‐fat diet‐treated *Apc^min/+^* mice activated MCP‐1/CCR2 axis and accelerated carcinogenesis. A‐C, Tumour numbers in both groups after FMT. D, The representative gross and histological appearance of intestinal tumours in both groups. E‐F, The expression levels of MCP‐1 and CCR2 in small intestine tumours of FMT‐H group were significantly higher than those in FMT‐C group. Scale bar: 50 μm. *P < .05, **P < .01, ***P < .001. FMT‐C, n = 4; FMT‐H, n = 4

To further confirm the important role of gut dysbiosis in promoting tumour development, faeces from HFD‐treated donors and faeces from control donors were transplanted to a new batch of *Apc^min/+^* mice, which were divided into FMT‐H group (transplantation of faecal microbiota from HFD group to a new batch of recipient *Apc^min/+^*mice) and FMT‐C group (transplantation of faecal microbiota from control group to a new batch of recipient *Apc^min/+^* mice). The total tumour number in FMT‐H group was significantly increased compared with that in the FMT‐C group (Figure 6A). FMT‐H increased the number of tumours in each segment of the intestine (Figure 6B). The numbers of all sizes of tumours were increased in FMT‐H group (Figure 6C). Histological analysis showed that intestinal carcinogenesis (HGD or intramucosal carcinoma) was detected in 75% mice of FMT‐H group, whereas adenomas with LGD were found only in 25% of *Apc^min/+^*mice in FMT‐C group, and no dysplastic adenomas were found in the remaining 75% mice (Figure 6D).

### 3.8 HFD‐induced dysbiosis could be transmitted to another batch of *Apc* ^min/+^ mice

**Figure 7:**
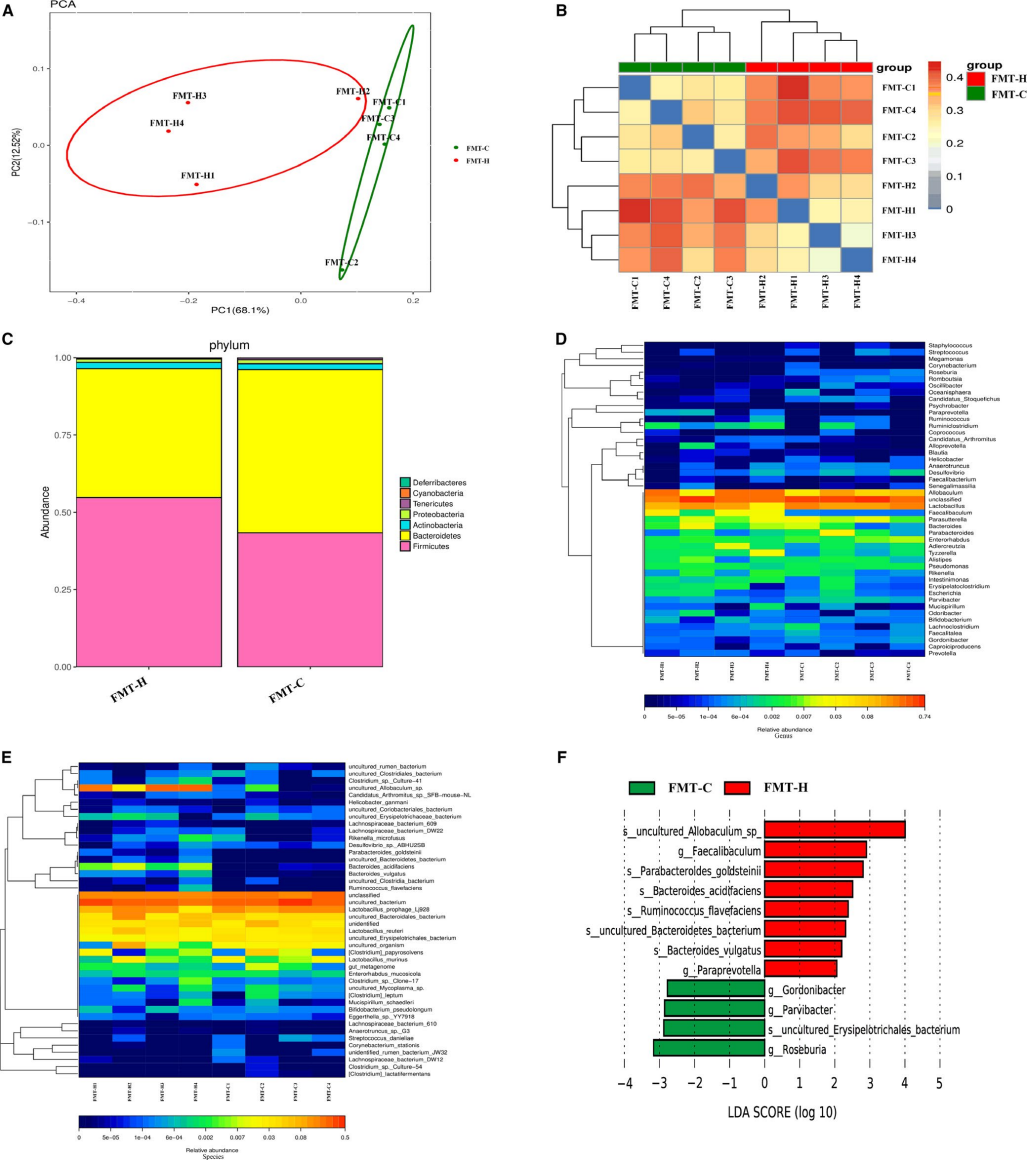
High‐fat diet‐induced dysbiosis could be transmitted to another batch of *Apc^min/+^* mice. A‐B, Principal component analysis (PCA) and beta‐diversity analysis showed clear difference in faecal microbiota clustering between FMT‐H group and FMT‐C group. C, Differences in microbial community composition between two groups at phylum level. D‐F, Heatmap and LEfSe results showed mice receiving the faecal microbiota from HFD‐fed donors had increased levels of opportunistic pathogens and decreased beneficial bacteria. HFD, high‐fat diet. FMT‐C (transplantation of faecal microbiota from control group to a new batch of recipient *Apc^min/+^*mice). FMT‐H (transplantation of faecal microbiota from HFD group to a new batch of recipient *Apc^min/+^*mice). FMT‐C, n = 4; FMT‐H, n = 4

PCA and beta‐diversity results showed significant differences in microbial community composition between FMT‐H group and FMT‐C group (Figure 7A‐B). At phylum level, compared with the FMT‐C group, the F/B ratio increased in the FMT‐H group (Figure 7C). At genus and species levels, the heatmap and LEfSe analysis of the microbial compositions showed that mice receiving the faecal microbiota from HFD‐fed donors had higher levels of opportunistic pathogens, such as *Paraprevotella, Parabacteroides*, *Odoribacter*,* Helicobacter*, *Bacteroides_acidifaciens* and *Bacteroides_vulgatus*, whereas the beneficial bacteria such as *Roseburia, Lactobacillus* and *Bifidobacterium* were relatively less abundant in the FMT‐H group compared with FMT‐C group (Figure 7D‐F).

### 3.9 Transfer of faecal microbiota from HFD‐fed *Apc^min/+^* mice also activated MCP‐1/CCR2 axis

The mRNA and protein expression levels of MCP‐1 and CCR2 increased significantly in tumours of FMT‐H group compared with that in FMT‐C group (Figure 6E‐F). These results further closely linked the HFD‐induced dysbiosis with the activation of MCP‐1/CCR2 axis, which might be a substantial factor in promoting intestinal carcinogenesis.

### 3.10 Antibiotics abolished HFD‐induced intestinal carcinogenesis

The total tumour number in HFD‐Abx mice was significantly decreased compared with that in the HFD group. Supplement with antibiotics in HFD group inhibited tumour development in the intestine. The number of tumours of various sizes was decreased in HFD‐Abx group (Figure S3A‐C). After the treatment of antibiotics cocktail, HGD was not found in the intestine (Figure S3D‐E). These data further supported that a strong link may exist between HFD‐induced gut dysbiosis and intestinal carcinogenesis.

## 4 DISCUSSION

**Figure 8:**
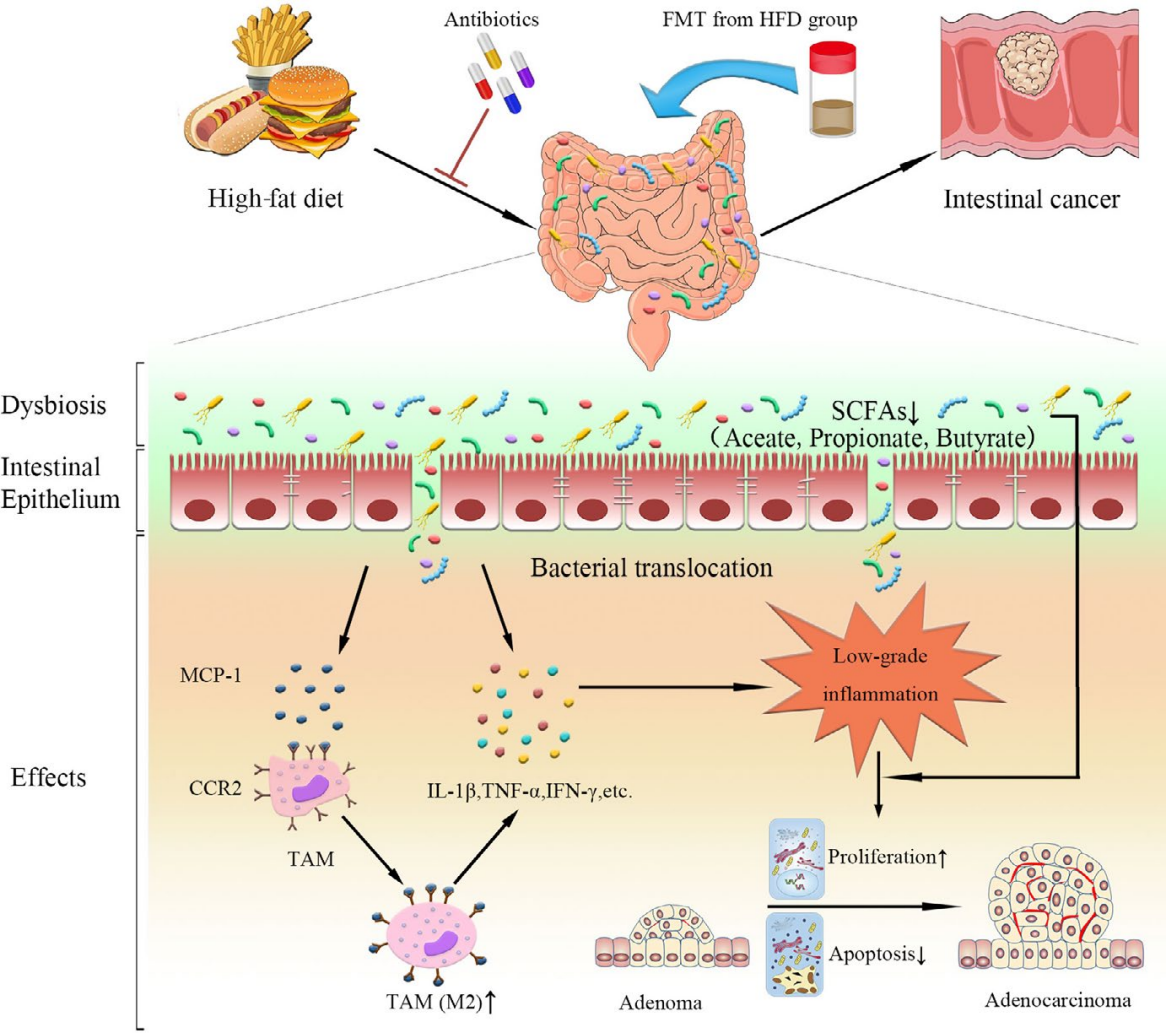
Schematic overview of high‐fat diet‐induced gut dysbiosis on intestinal carcinogenesis. HFD intake significantly altered the gut microbial composition and caused dysbiosis in *Apc^min/+^* mice. Disrupted intestinal barrier favoured the bacterial translocation. The dysbiosis activated the MCP‐1/CCR2 signalling axis to recruit and polarize M2 TAMs. In addition, these changes increased the release of inflammatory mediators and also reduced the SCFAs production. These cancer‐promoting processes promoted tumour cell proliferation and inhibited apoptosis to facilitate colorectal neoplastic progression. Antibiotics cocktail treatment could reverse HFD‐induced intestinal carcinogenesis, whereas transfer of faecal microbiota from HFD‐fed mice to another batch of *Apc^min/+^* mice in the absence of HFD could activate MCP‐1/CCR2 axis and promote the development of adenoma‐adenocarcinoma sequence. HFD, high‐fat diet; FMT, faecal microbiota transplantation; MCP‐1, monocyte chemoattractant protein 1; CCR2, CC chemokine receptor 2; TAMs, tumour‐associated macrophages; SCFAs, short‐chain fatty acids; IL‐1β, interleukin‐1β; TNF‐α, tumour necrosis factor‐α; IFN‐γ, interferon‐γ

Substantial evidence suggested that individuals who have adopted a ‘western‐style’ diet with high‐fat content have a higher risk of developing CRC.^33^, ^34^ Although intensive investigations have been conducted, identifying molecular mechanisms that how HFD promoted CRC development remained a challenge. The present study combined diet, microbiota and CRC to better investigate the role of gut microbiota regulating innate immunity in the progression of intestinal adenoma. Here we demonstrated that HFD was linked to higher incidence of AN, and we also found the close relationship between HFD and MCP‐1/CCR2 axis in patients with CRC. HFD‐induced dysbiosis promoted tumour progression in the *Apc^min/+^* mouse model independently of weight gain. The consumption of HFD mediates a shift in the composition of the gut microbiota and eventually caused the increased abundance of pathogens and decreased of probiotics. It was highlighted that HFD reduced the abundance of SCFAs‐producing bacteria and the content of caecal SCFAs. The structural shift of microbial community activated the MCP‐1/CCR2 axis and augmented M2 TAMs recruitment and polarization. Furthermore, the transfer of faecal microbiota from HFD‐fed *Apc^min/+^* mice to a new batch of *Apc^min/+^* mice was sufficient to transmit tumorigenicity in the absence of HFD; meanwhile, antibiotics treatment eliminated the tumorigenesis caused by dysbiosis (Figure 8). These results that we have identified therefore assist in our understanding of how HFD promoted intestinal carcinogenesis.

There are persuasive arguments to consider HFD and microbiota dysbiosis as key risk factors of CRC.^13^, ^35^ About 10^14^ microbes populated in the human gut,^36^ and many bacteria have been proved of having a pro‐tumorigenic effect, such as *Fusobacterium nucleatum*, *Escherichia coli*, *Bacteroides fragilis* and *Peptostreptococcus anaerobius*.^37^ It is well known that HFD is the main cause of obesity. The gut microbiota is also reported as a contributing factor to the pathophysiology of obesity, and the obese microbiome can be more effective in harvesting energy from diet.^38^, ^39^ However, in the development of CRC, it is still unclear which one is the main driver between HFD‐induced dysbiosis and obesity. In our study, HFD‐induced dysbiosis had tumorigenic effects but cannot promote weight gain. This is in line with previous reports that the alternation of gut microbiota resulting from HFD has a greater impact on the disease independent of obesity.^13^, ^40^ FMT experiments in germ‐free mice have shown pro‐tumorigenic effects of the stools from patients with CRC.^41^ To verify that HFD‐induced dysbiosis is carcinogenic, we also performed FMT for subsequent studies. We found that the dysbiosis from HFD‐treated mice could be transmitted and significantly promoted carcinogenesis. Antibiotics may be effective in the treatment of CRC Johnson et al^42^ found that the biofilm in antibiotic‐treated CRC patients was decreased, which protected the colon mucous barrier. Antibiotic treatment was effective to deplete the gut microbiota to inhibit carcinogenesis.^43^, ^44^ Our research also confirmed the protective role of antibiotics in the progression of intestinal tumours. Collectively, all results verified the importance of dysbiosis in promoting CRC development.

The carbohydrate that escaped digestion in the small intestine is fermented to SCFAs by microbiota.^45^ SCFAs mainly include acetate, propionate and butyrate, and have anti‐inflammatory and antineoplastic properties, particularly butyrate, which is the preferred energy source for colonocytes.^23^ Related clinical studies revealed that patients with advanced CRC had diminished butyrate‐producing bacteria and lower levels of SCFAs when compared with healthy controls.^46^, ^47^, ^48^ Our study showed that the abundance of SCFAs‐producing bacteria (*Roseburia*, *Barnesiella*, *Lachnospiraceae bacterium‐A4* and *Lachnospiraceae bacterium‐COE1*) was reduced in HFD group. Interestingly, the level of caecal SCFAs (acetate, propionate and butyrate) was decreased in different degrees after HFD treatment. However, it is controversial whether HFD can reduce the level of SCFAs. It is reported that HFD increased the content of acetic acid,^49^ which is contrary to the results of other studies.^13^, ^50^, ^51^ The observed results highlighted that the production of SCFAs was inextricably linked to gut microbiota, and high fibre diet may have certain preventive effect on the occurrence of CRC.^47^

Chemokines are structurally divided into CC, CXC, CX3C and C chemokines, and are well known for the ability to induce migration or chemotaxis of immune cells and involved in the pathogenesis cancers.^52^ MCP‐1 is one of the key members of the CC chemokine family and regulate the migration of innate and adaptive immune cells after binding to the cognate receptor CCR2.^53^ According to the previous reports, MCP‐1 expression was markedly up‐regulated in colorectal adenoma with mild‐moderate dysplasia and HGD than in normal mucosa and was associated with a negative prognosis of patients with CRC.^12^, ^54^ Moreover, data also identified that overexpression of MCP‐1 was correlated with colon cancer liver metastasis.^55^ The circulating monocytes were recruited by MCP‐1 to the tumour microenvironment and differentiate into TAMs which produced a large number of lymphangiogenic growth factors, cytokines and proteases to promote tumour cell proliferation and metastasis.^56^, ^57^ The ability of TAMs to adapt to the environment has led to the identification of two main phenotypes, one is the classically activated M1‐type TAMs which exhibit a proinflammatory effect, and with the progression of the tumours, M1‐type TAMs switch to more immunosuppressive M2‐type TAMs, which promote tumour growth, survival and metastasis.^58^ In our study, MCP‐1/CCR2 axis was activated by HFD‐induced dysbiosis, which promoted the recruitment and polarization of M2 TAMs. Remarkably, the transfer of faeces from HFD‐fed mice could activate MCP‐1/CCR2 axis. We also showed that HFD increased the expression of MCP‐1, CCR2 and CD163 in human CRC specimens. Some studies have shown that in the stage of CRC, higher expression of these proteins could increase the invasion and metastasis of CRC cells and was associated with shorter overall survival.^11^, ^29^, ^55^, ^59^, ^60^, ^61^ However, our research mainly focused on the different expression of these proteins during the carcinogenesis induced by HFD. We also selected patients with no significant differences in pathological characteristics to avoid different protein expressions due to different CRC aggressiveness. Hence, these data strongly proved the close relationship between the HFD‐induced dysbiosis and MCP‐1/CCR2 axis in the progression of intestinal tumours.

Some potential limitations of the current study should be noted. For instance, a prospective cohort study could better investigate the association between HFD and gut microbiota dysbiosis in intestinal carcinogenesis. However, Ludwig et al^62^ found that prospective studies evaluating dietary interventions for chronic diseases have far greater challenges in terms of consistency, quality control, confounding and interpretation. In addition, for our research, different types of red meat (pork, beef or lamb) contain different ingredients, such as saturated fatty acids and haem, which may cause a significant difference in the gut microbiota.^63^, ^64^ Recently, a 6‐month randomised controlled‐feeding trial by Wan et al have indirectly explored the effects of HFD on human gut microbiota. They found that higher‐fat diet could increase the abundance of *Alistipes* and *Bacteroides*, and decrease the concentrations of total SCFAs.^51^ These data were consistent with the results of our animal studies. We will conduct a prospective cohort study to better verify our results. It is necessary to collect more comprehensive information and strictly limit the factors such as the age, gender, type of red meat and other food categories.

Altogether, these findings revealed that how HFD‐induced dysbiosis contributed to the intestinal adenoma‐adenocarcinoma sequence. The dysbiosis was associated with the increased proliferation and decreased apoptosis of intestinal tumour and reduced the content of SCFAs. More importantly, it might activate the MCP‐1/CCR2 axis and then promote the recruitment and polarization of M2 TAMs. Thus, regulating microbiota and MCP‐1/CCR2 axis may represent an impactful strategy in CRC therapeutics. Meanwhile, further research will be required to verify the personalized dietary interventions targeting the gut microbiota for CRC prevention.

## CONFLICT OF INTEREST

The authors declare no conflict of interest.

## AUTHOR CONTRIBUTIONS

TYL and ZXG contributed equally to this work; TYL, ZXG, LL, WXD, SNW, XLS and HLC were involved in the experiments and data analysis; TYL, ZXG, MQX and HLC were involved in writing the manuscript; CY, BMW and HLC were involved in the study design and the critical review and revision of the manuscript; and all authors who contributed to the design and writing of the paper agreed with the final version and the content of the manuscript.

## Data Availability

The data that support the findings of this study are available from the corresponding author upon reasonable request.
