# Supplementary figures and images for: High‐fat diet‐induced dysbiosis mediates MCP‐1/CCR2 axis‐dependent M2 macrophage polarization and promotes intestinal adenoma‐adenocarcinoma sequence
Source: J Cell Mol Med. 2020 Jan 19;24(4):2648–62. doi: 10.1111/jcmm.14984 (PMC7028862; doi:10.1111/jcmm.14984)

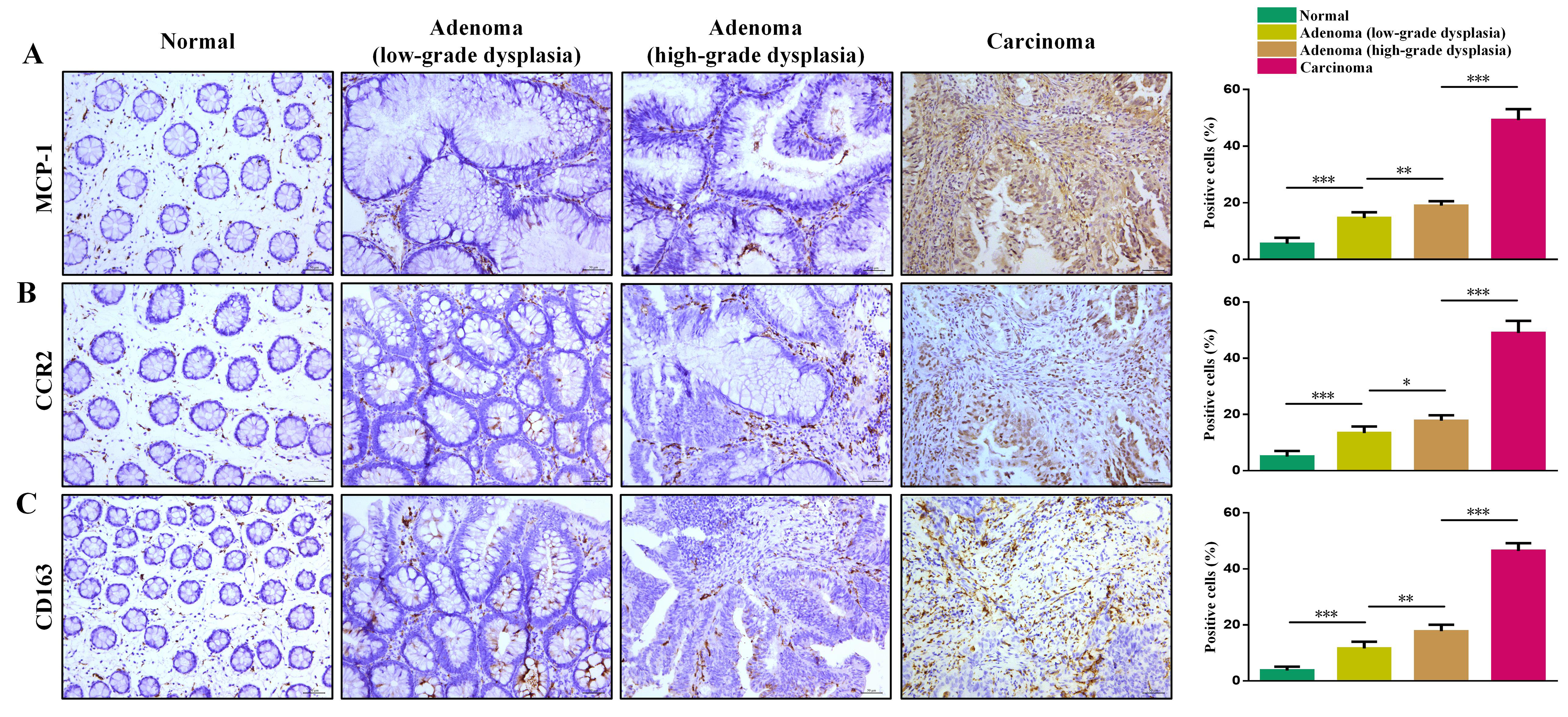

Supplement: Supplementary file 1 [file JCMM-24-2648-s001.tif]

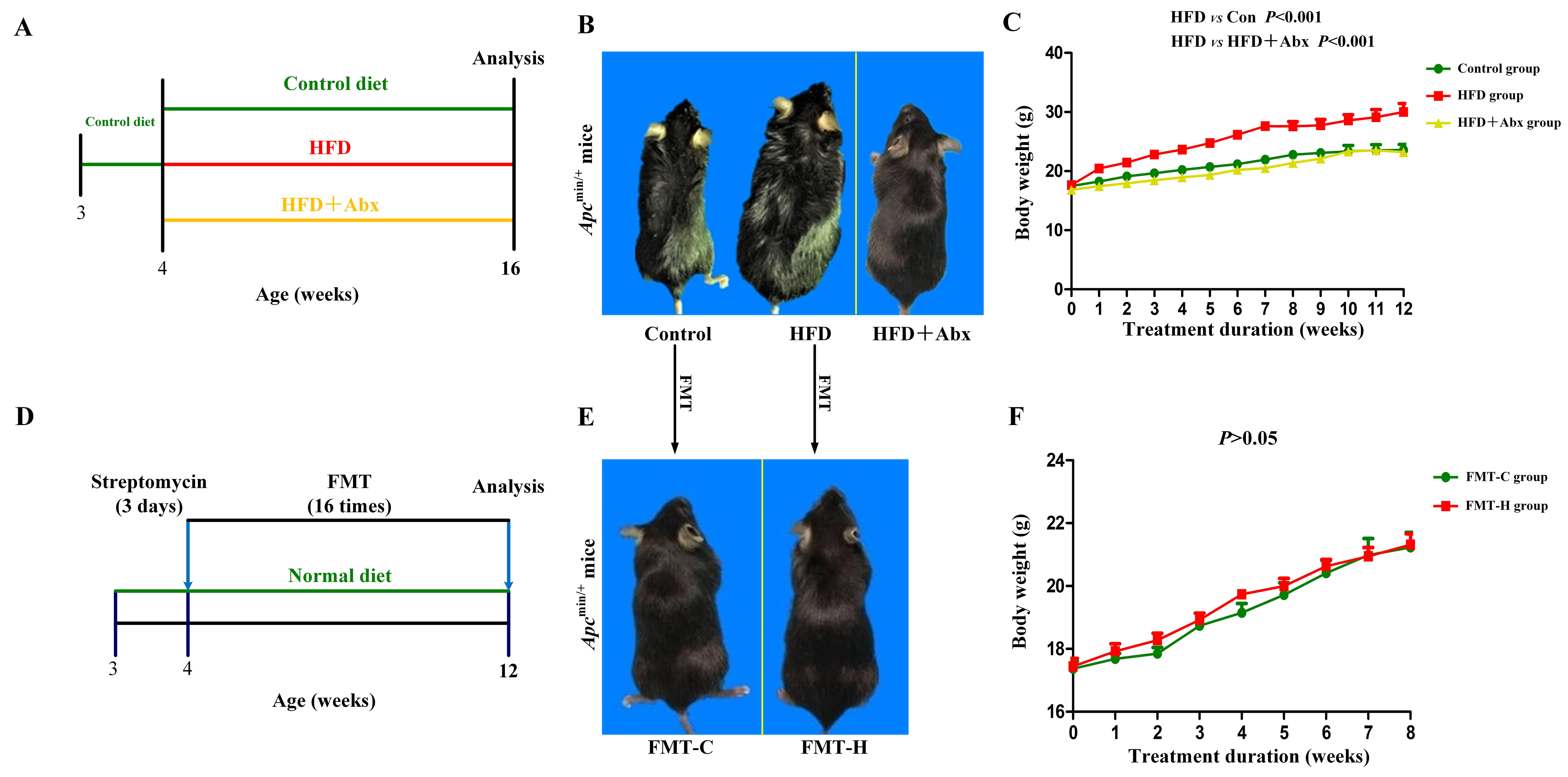

Supplement: Supplementary file 2 [file JCMM-24-2648-s002.tif]

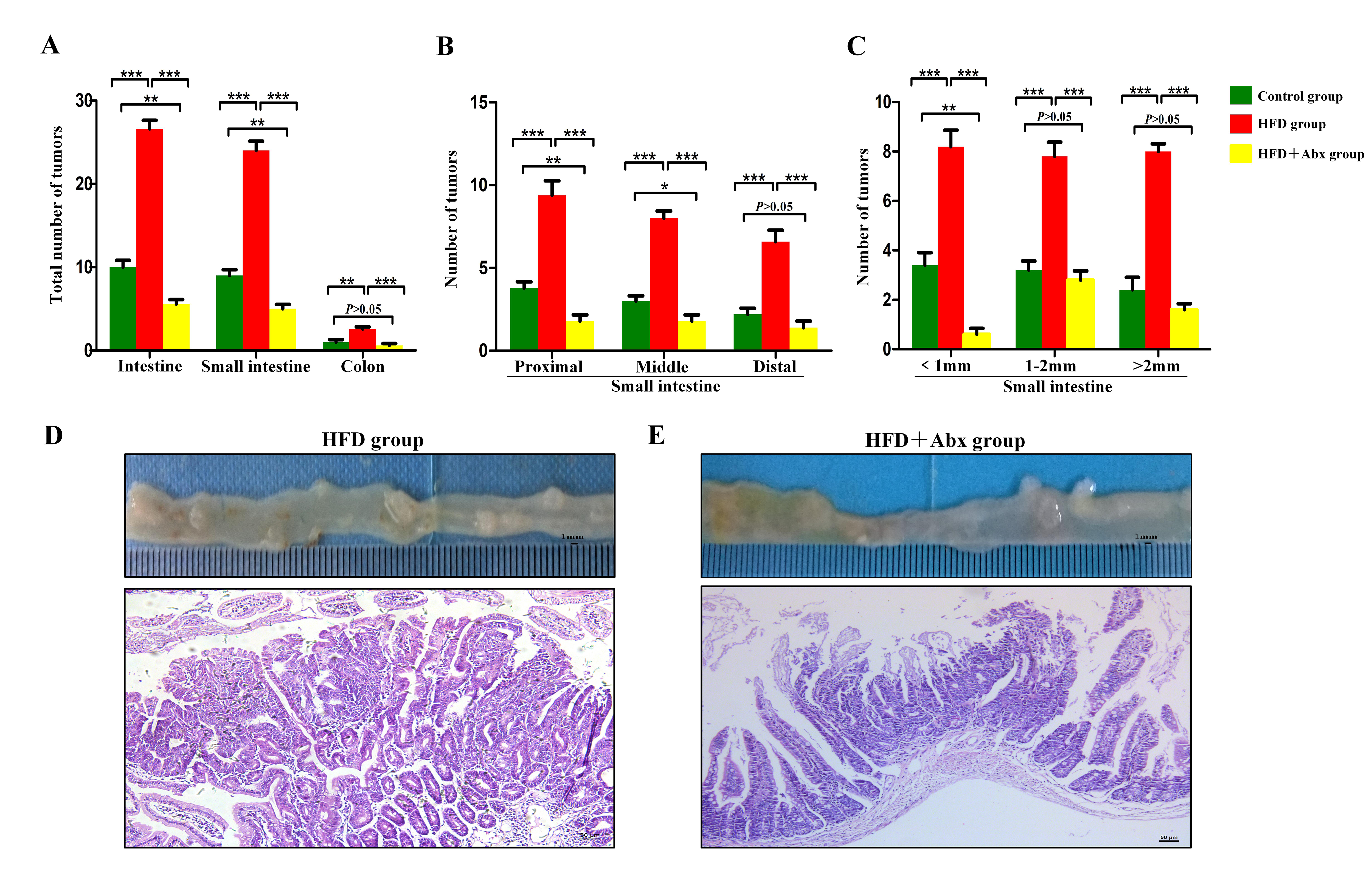

Supplement: Supplementary file 3 [file JCMM-24-2648-s003.tif]
