# Supplementary material for: High‐fat diet‐induced dysbiosis mediates MCP‐1/CCR2 axis‐dependent M2 macrophage polarization and promotes intestinal adenoma‐adenocarcinoma sequence
Source: J Cell Mol Med. 2020 Jan 19;24(4):2648–62. doi: 10.1111/jcmm.14984 (PMC7028862; doi:10.1111/jcmm.14984)
Supplement: Supplementary file 4 [file JCMM-24-2648-s004.docx]

**Table S1 Composition of high fat diet and control diet**

|  | **High fat diet** | **Control diet** |
| --- | --- | --- |
| **Protein,%** | 20 | 20 |
| **Carbohydrate,%** | 20 | 64 |
| **Fat,%** | 60 | 16 |
| **Component, kcal/kg** | | |
| **Casein** | 1034 | 800 |
| **Cystine** | 16 | 12 |
| **Maltodextrin** | 646 | 528 |
| **Sucrose** | 356 | 400 |
| **Soybean oil** | 291 | 630 |
| **Lard oil** | 2849 | 0 |
| **Vitamin mixture** | 52 | 40 |

**Table S2 The Oligonucleotide primers for target genes**

| Primers | Sequence |
| --- | --- |
| GAPDH  (Glyceraldehyde-3-phosphatedehydrogenase) | Forward:5’- GGAGAAACCTGCCAAGTATG-3’  Reverse:5’- TGGGAGTTGCTGTTGAAGTC-3’ |
| IL-1β  (Interleukin-1β) | Forward:5’- GTGGCTGTGGAGAAGCTGTG-3’  Reverse:5’- GAAGGTCCACGGGAAAGACAC-3’ |
| TNF-α  (Tumor necrosis factor-α) | Forward:5’- ACTCCAGGCGGTGCCTATG-3’  Reverse:5’- GAGCGTGGTGGCCCCT-3’ |
| IFN-γ  (Interferon-γ) | Forward:5’-GCATCTTGGCTTTGCAGCT-3’  Reverse:5’-CCTTTTTCGCCTTGCTGTTG-3’ |
| MCP-1  (Monocyte chemoattractant protein 1) | Forward:5’-TTAAAAACCTGGATCGGAACCAA-3’  Reverse:5’- CTTTGGCTATGGGCTTCCAGTC-3’ |
| CCR2  (CC chemokine receptor 2) | Forward:5’- CACGAAGTATCCAAGAGCTT-3’  Reverse:5’- CATGCTCTTCAGCTTTTTAC-3’ |
| iNOS  (inducible nitric oxide synthase) | Forward:5’- GCAAACATCACATTCAGATCCC-3’  Reverse:5’- TCAGCCTCATGGTAAACACG-3’ |
| MR  (macrophage mannose receptors) | Forward:5’-GGTGCTACTCCGAACAACAG-3’  Reverse:5’- ACCGTGGCTGAAAGTTCCT-3’ |
| Arg-1  (Arginase 1) | Forward:5’-TGGCTTGCGAGACGTAGAC-3’  Reverse:5’-GCTCAGGTGAATCGGCCTTTT-3’ |

**Table S3 Baseline characteristics of selected patients with colorectal cancer by diet category**

| **Characteristics** | **High fat diet (n=15)** | **Control diet (n=15)** | ***P*** |
| --- | --- | --- | --- |
| **Male/Female** | 8/7 | 8/7 | 1.000 |
| **Age (years)** | 62 (29-75) | 68 (47-76) | 0.095 |
| **BMI** |  |  | 1.000 |
| **≥25** | 6 | 6 |  |
| **<25** | 9 | 9 |  |
| **History of adenoma** | 2 | 0 | 0.483 |
| **Family history** |  |  | 0.598 |
| **Cancer** | 1 | 2 | 1.000 |
| **Adenoma** | 0 | 1 | 1.000 |
| **Smoking** |  |  | 0.522 |
| **Current** | 5 | 2 | 0.390 |
| **Former** | 1 | 2 | 1.000 |
| **Never** | 9 | 11 | 0.700 |
| **Alcohol consumption**  **(≥3 times/week)** | 2 | 3 | 1.000 |
| **Tea consumption**  **(≥3 times/week)** | 5 | 4 | 1.000 |
| **Coffee consumption**  **(≥3 times/week)** | 1 | 1 | 1.000 |
| **TNM stage II/III/IV** | 3/5/7 | 3/5/7 | 1.000 |
