# Supplementary material for: High‐fat diet‐induced dysbiosis mediates MCP‐1/CCR2 axis‐dependent M2 macrophage polarization and promotes intestinal adenoma‐adenocarcinoma sequence
Source: J Cell Mol Med. 2020 Jan 19;24(4):2648–62. doi: 10.1111/jcmm.14984 (PMC7028862; doi:10.1111/jcmm.14984)
Supplement: Supplementary file 5 [file JCMM-24-2648-s005.docx]

**Figure S1** The expression of MCP-1, CCR2 and CD163 in human colonic tissues showed a gradual increase during intestinal normal-adenoma-adenocarcinoma sequence. Scale bar: 50μm. *, *P*< 0.05, **, *P*< 0.01, ***, *P*< 0.001. n = 10 for each group.

**Figure S2** The experimental flow and body weight change during the study. A, HFD, control diet and HFD+Abx were administered to *Apc^min/+^* mice for 12 weeks until sacrifice. B-C, The body weight of HFD group was significantly higher than that of control group and HFD+Abx group throughout the treatment period (*P*<0.001). D, *Apc^min/+^* mice were gavaged with streptomycin (20 mg) for 3 days and then were inoculated with faecal microbiota for 8 weeks. E-F, No significant difference in body weight between the FMT-H group and FMT-C group was evident throughout the treatment period (*P*>0.05). Control, n=7; HFD, n=8; HFD+Abx, n=8; FMT-C, n=4; FMT-H, n=4.

**Figure S3** Antibiotics cocktail treatment inhibited intestinal tumour development. Antibiotics cocktail treatment inhibited intestinal tumour development. A, The total tumour numbers in the small intestine and colon in the control, HFD and HFD+Abx group. B-C, The number of tumours in each section and different sizes of the small intestine in three groups were listed. D-E, The representative gross and histological appearance of intestinal tumours from the HFD group and HFD+Abx group were shown. Antibiotics cocktail inhibited HFD-induced intestinal carcinogenesis. Scale bar: 50μm. *, *P*< 0.05, **, *P*< 0.01, ***, *P*< 0.001. HFD, high-fat diet. Abx, antibiotics. Control, n=7; HFD, n=8; HFD+Abx, n=8.
